# Multifunctional Zn and Ag co-doped bioactive glass nanoparticles for bone therapeutic and regeneration

**DOI:** 10.1038/s41598-023-34042-w

**Published:** 2023-04-25

**Authors:** Parichart Naruphontjirakul, Pimpikar Kanchanadumkerng, Pakatip Ruenraroengsak

**Affiliations:** 1 Biological Engineering Program, Faculty of Engineering, King Mongkut’s University of Technology Thonburi, Bangkok, 10140 Thailand; 2 Department of Food Chemistry, Faculty of Pharmacy, Mahidol University, Bangkok, 10400 Thailand; 3 Division of Pharmaceutical Technology, Department of Pharmacy, Faculty of Pharmacy, Mahidol University, Bangkok, 10400 Thailand

**Keywords:** Biomaterials, Nanoscale materials, Nanoparticles

## Abstract

Bone cancer has traditionally been treated using surgery, radiotherapy, and/or chemotherapy. The nonspecific distribution of chemotherapy and implantable infections are significant risk factors for the failure of the bone to heal. Multifunctional zinc and silver co-doped bioactive glass nanoparticles (yAg–xZn-BGNPs) with a diameter of 150 ± 30 nm were successfully synthesized using modified sol–gel and two-step post-functionalization processes, tailored to provide antibacterial and anticancer activity whilst maintaining osteogenesis ability. Co-doped BGNPs with Zn and Ag did not significantly alter physicochemical properties, including size, morphology, glass network, and amorphous nature. Apatite-like layer was observed on the surface of yAg–xZn-BGNPs and resorbed in the simulated body fluid solution, which could increase their bioactivity. Human fetal osteoblast cell line (hFOB 1.19) treated with particles showed calcified tissue formation and alkaline phosphatase activity in the absence of osteogenic supplements in vitro, especially with 0.5Ag–1Zn-BGNPs. Moreover, these particles preferentially disrupted the metabolic activity of bone cancer cells (MG-63) and had an antibacterial effect against *B. subtilis, E. coli*, and *S. aureus* via the disc diffusion method. This novel 0.5Ag–1Zn-BGNP and 1Ag–1Zn-BGNPs, with wide-ranging ability to stimulate bone regeneration, to inhibit bone cancer cell proliferation, and to prevent bacterial growth properties, may provide a feasible strategy for bone cancer treatment. The 0.5Ag–1Zn-BGNPs and 1Ag–1Zn-BGNPs can be applied for the preparation of scaffolds or filler composites using in bone tissue engineering.

## Introduction

Cancer is the second-leading cause of death worldwide, causing approximately 10 million deaths in 2020. Bone cancer (malignant bone tumors) can occur in both children and adults, and is divided into 2 types: primary bone cancer, caused by the dysfunctional bone cell itself (osteosarcoma), and secondary bone cancer (metastasis), caused by the spread of cancer cells to the bone in the late stages of almost all cancers^1–3^. The typical approaches for treating bone cancer are surgery, radiation, chemotherapy, and targeted therapy, based on evaluation of a treatment’s benefits against its potential side effects, and depending on the type and stage of bone cancer. The main purpose of surgery is to remove part of the cancerous area of bone and to reconstruct the bone. However, it's usually combined with the other treatments. The combination of surgery and neoadjuvant or adjuvant chemotherapy increased 5-year survival rates of patients with localized bone cancers up to 70%, but only up to 20% for patients with recurrent or metastatic bone cancer^4^. However, the adverse side effects of chemotherapy caused both healthy and cancerous cell damage. In addition, infections during bone cancer treatment have the potential to lead to failure of reconstructions. The rate of postsurgical infection was reported ranging from 12 to 47%^5^. The most commonly Gram-positive bacteria associated with biofilm-related infections is *Staphylococcus aureus*^6^*.* Biomaterials containing antimicrobial agents or release therapeutics within the local microenvironment have been developed. However, there are a limited number of studies with biomaterials containing bone regeneration, anticancer, and local antibiotic delivery capabilities. After bone cancer therapy, the next concern was bone defects. Thus, multi-purpose biomaterials through either local or systemic administration have become an essential research challenge^7,8^. Non-specificity of radiation therapy and chemotherapy destroys both normal and cancer cells, causing severe adverse side effects and significantly limiting their clinical applications^7^.

To improve the efficacy of cancer treatment and overcome drug resistance, nanoparticles (NPs) have been introduced as nanocarriers in cancer treatment research because of their unique properties, including high specific surface area and surface-to-volume ratio^9^. Size and morphology of nanoparticles play a critical role in the internalization and localization of cancer cells. Moreover, leaky tumor vasculature in combination with a poorly-developed lymphatic drainage system leads to selective penetration and accumulation of the NPs through the passive targeting^10^. In addition, NPs were modified with specific ligands such as monoclonal antibodies, peptides, and aptamers to specific target the bone cells and tumors^11^.

Bioactive glass nanoparticles (BGNPs) have attracted extensive attention in the field of drug delivery systems due to their biodegradability, bioactivity, and biocompatibility. BGNPs have great potential to induce new bone formation through hydroxyapatite formation and protect infection through the released metallic ions, as well as exhibiting anticancer activity^12^. The desirable properties of BGNPs are based on their composition. Sol–gel-derived BGNPs, with high porosity, controlled particle size uniformity, and high purity, have an amorphous nature; therefore, various therapeutic cations can be incorporated into the glass network^13^. Mesoporous BGNPs have often been used in cancer treatment applications owing to their large pore size and area for loading therapeutic agents^14–17^.

In a previous study, strontium (Sr) was introduced into a glass network as a modifier cation by partially replacing CaO with SrO^18^. Sr-containing BGNPs (Sr-BGNPs) have the ability to enhance bone formation by stimulating osteoblast activity and inhibit bone resorption by inhibiting osteoclast activity^19,20^. Zinc (Zn) was doped into BGNPs to improve bioavailability properties, including inhibiting osteoclastic bone resorption by inhibiting osteoclast-like cell formation^21^, inducing osteoblastic bone formation by activating osteoblast-like cell differentiation^22^, preventing bacterial infections^23,24^, and exhibiting anticancer activity^25^. Silver (Ag) has been incorporated into BGNPs to enhance bioactivity^26^. Ag functioned as a network modifier in a glass network, leading to a leaky network structure that could be easily degraded^27^. Moreover, the Ag released from BGNPs disrupted bacterial function^28,29^. However, the impact of either Zn or Ag compared to Sr-containing BGNPs on bone cells has not been reported yet.

The dense BGNPs sustain the resorption, resulting in a prolonged therapeutic effect. The hypothesis of this study was to promote the synergistic influence of Zn and Ag co-doping BGNPs on stimulating bone regeneration, killing bone cancer, and preventing bone infection. The aims of this research were to develop multifunctional BGNPs with a combination of bone regeneration, antibacterial, and anticancer properties by incorporating Zn and Ag into a SiO_2_–CaO–SrO ternary glass system and to investigate the combined effect of Sr, Zn, and Ag therapeutic ions in dense BGNPs. The multifunctional Zn- and Ag-doped sol–gel-derived BGNPs were synthesized using the sol–gel technique and two-step post-functionalization. The size and morphology of the particles were evaluated using Scanning Electron Microscopy (SEM). The glass structure and amorphous nature were investigated using Fourier Transform Infrared (FTIR) and an X-ray Diffractometer (XRD). The ions released through particle degradation were evaluated using Transmission Electron Microscopy (TEM) and Energy-dispersive X-ray Spectroscopy (EDX-SEM). The effect of BGNPs on the viability of MC3T3-E1, hFOB 1.19, and MG-63 cell lines was compared using MTT assay. Non-tumor and cancerous bone cells were selected to investigate the anticancer activity of BGNPs. The antibacterial activity of prepared BGNPs against *Bacillus subtilis*, *Escherichia coli*, and *Staphylococcus aureus* was assessed using the paper disc diffusion method.

## Results and discussion

**Figure 1:**
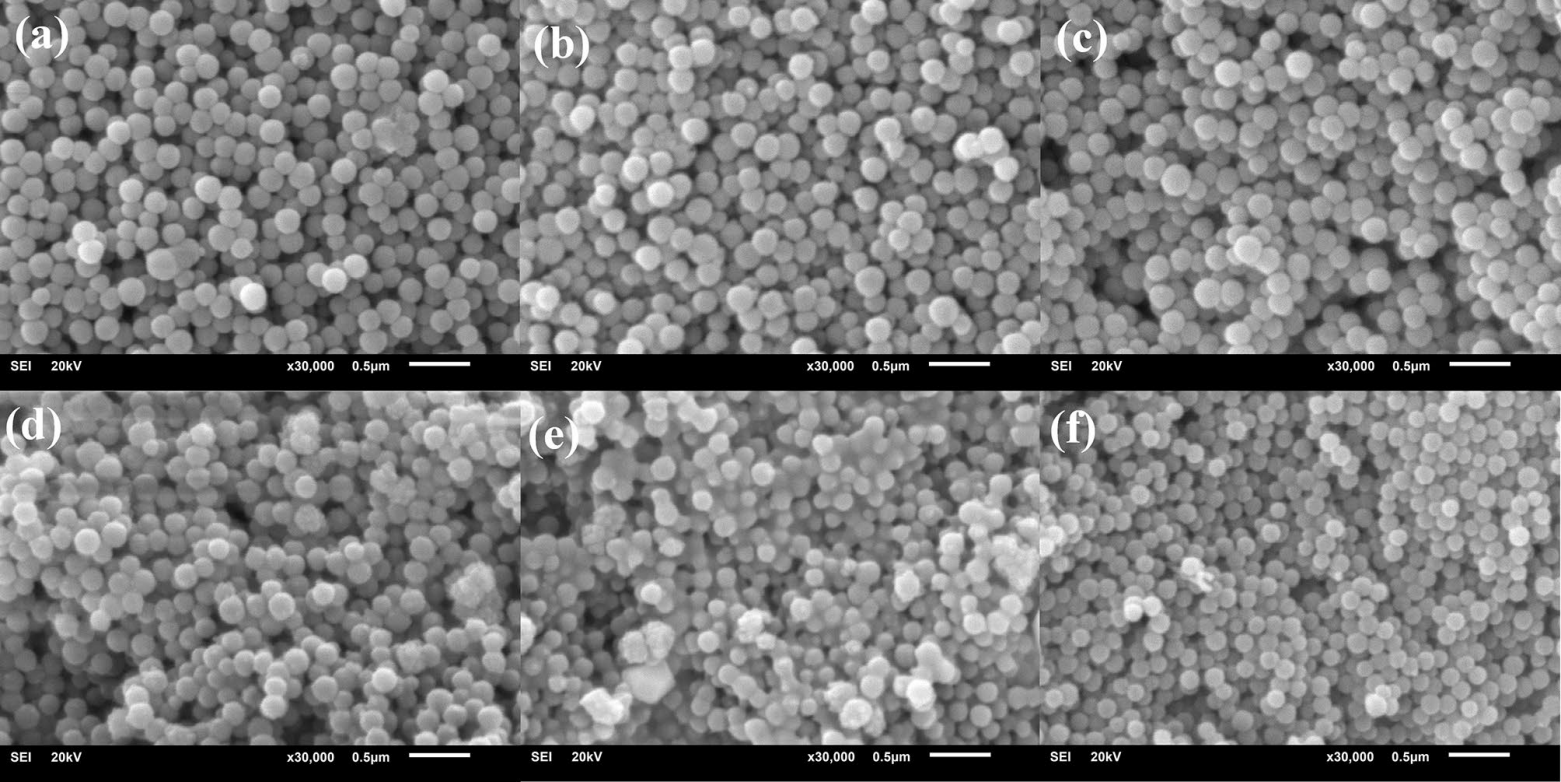
Bright field SEM images of sol–gel derived BGNPs (**a**) 0Ag–0Zn-BGNPs, (**b**) 0Ag–1Zn-BGNPs, (**c**) 0.5Ag–1Zn-BGNPs, (**d**) 1Ag–1Zn-BGNPs, (**e**) 1.5Ag–1Zn-BGNPs, and (**f**) 1.5Ag–1.5Zn-BGNPs (operating at 20 kV and magnification 30 kX, scale bars = 500 nm).

Dense monodispersed BGNPs with a diameter range of 150 ± 30 nm were successfully synthesized through the sol–gel and two-step post-functionalization processes as shown in Table 1. The zeta potential of 0Ag–0Zn-BGNPs was -33.4 ± 6.2 mV. The zeta potential shifted to the positive when the Zn and Ag were incorporated. This might be due to an increase in the overall amount of network modifiers in the glass network. Previous work reported that Zn incorporated into the silica network with high binding affinity compared to Ca and Sr (the alkaline earth metals); therefore, the two-step post-functionalization method was used to incorporate cations into the glass structure. In this study, 0Ag–0Zn-BGNPs with a nominal ratio of Si:Ca/Sr 1:2 were fabricated first, followed by the second post-functionalization with Zn and Ag (Table 3). The particles were monodispersed, with a spherical structure homogeneous in size and shape, as shown in Fig. 1. However, the particle size of 1.5Ag–1.5Zn-BGNPs was a bit smaller than other compositions. This may indicate that the addition of Zn at the nominal ratio of 0, 1, and 1.5, and Ag at 0, 0.5, 1.0, and 1.5, did not significantly affect the morphology and homogeneity of the incorporated particles.

**Table 1:** Particle size and surface charge of BGNPs.

	Size (nm)	PDI	Zeta potential (mV)
0Ag–0Zn-BGNPs	167.13 ± 2.73	0.25 ± 0.06	− 33.4 ± 6.2
0Ag–1Zn-BGNPs	167.47 ± 6.85	0.29 ± 0.03	− 25.7 ± 5.8
0.5Ag–1Zn-BGNPs	170.20 ± 6.39	0.49 ± 0.06	− 13.9 ± 2.4
1Ag–1Zn-BGNPs	170.53 ± 11.13	0.35 ± 0.03	− 16.7 ± 1.8
1.5Ag–1Zn-BGNPs	161.60 ± 4.94	0.37 ± 0.08	− 17.0 ± 1.8
1.5Ag–1.5Zn-BGNPs	147.83 ± 9.14	0.23 ± 0.03	− 16.4 ± 1.3

The elements in the composition of the prepared BGNPs were measured using X-ray fluorescence (XRF). The XRF results demonstrated the incorporation of Ca, Sr, Zn, and Ag through the two-step post-functionalization. The total amount of Ca and Sr in BGNPs decreased significantly—from approximately 53 to 18 wt%—when Zn and Ag were doped, as shown in Table 2. These results indicated that Zn and Ag had a high binding affinity to the silica network compared to Ca and Sr. When the nominal ratio of Ag increased, the amount of Ag in the composition increased, while the amount of Zn in the composition decreased. The total amount of Zn and Ag of 1.5Ag–1.5Zn-BGNPs was lower than that of 0, 1, and 1.5Ag–1Zn-BGNPs, implying not all doped Zn and Ag could incorporate into the BGNPs.

**Table 2:** Elemental compositions of BGNPs (wt%).

	Si	Ca	Sr	Zn	Ag
0Ag–0Zn-BGNPs	46.48 ± 0.41	17.52 ± 0.15	35.97 ± 0.39		
0Ag–1Zn-BGNPs	31.58 ± 0.59	5.62 ± 0.09	21.40 ± 0.26	41.40 ± 0.26	
0.5Ag–1Zn-BGNPs	28.14 ± 0.43	5.14 ± 0.04	16.95 ± 0.14	27.94 ± 0.17	21.83 ± 0.12
1Ag–1Zn-BGNPs	31.32 ± 0.61	4.96 ± 0.04	13.77 ± 0.16	20.31 ± 0.16	29.64 ± 0.29
1.5Ag–1Zn-BGNPs	31.10 ± 0.79	4.33 ± 0.19	14.03 ± 0.08	16.13 ± 0.13	34.41 ± 0.43
1.5Ag–1.5Zn-BGNPs	28.41 ± 1.00	6.93 ± 0.21	22.30 ± 0.28	18.58 ± 0.23	23.78 ± 0.36

**Figure 2:**
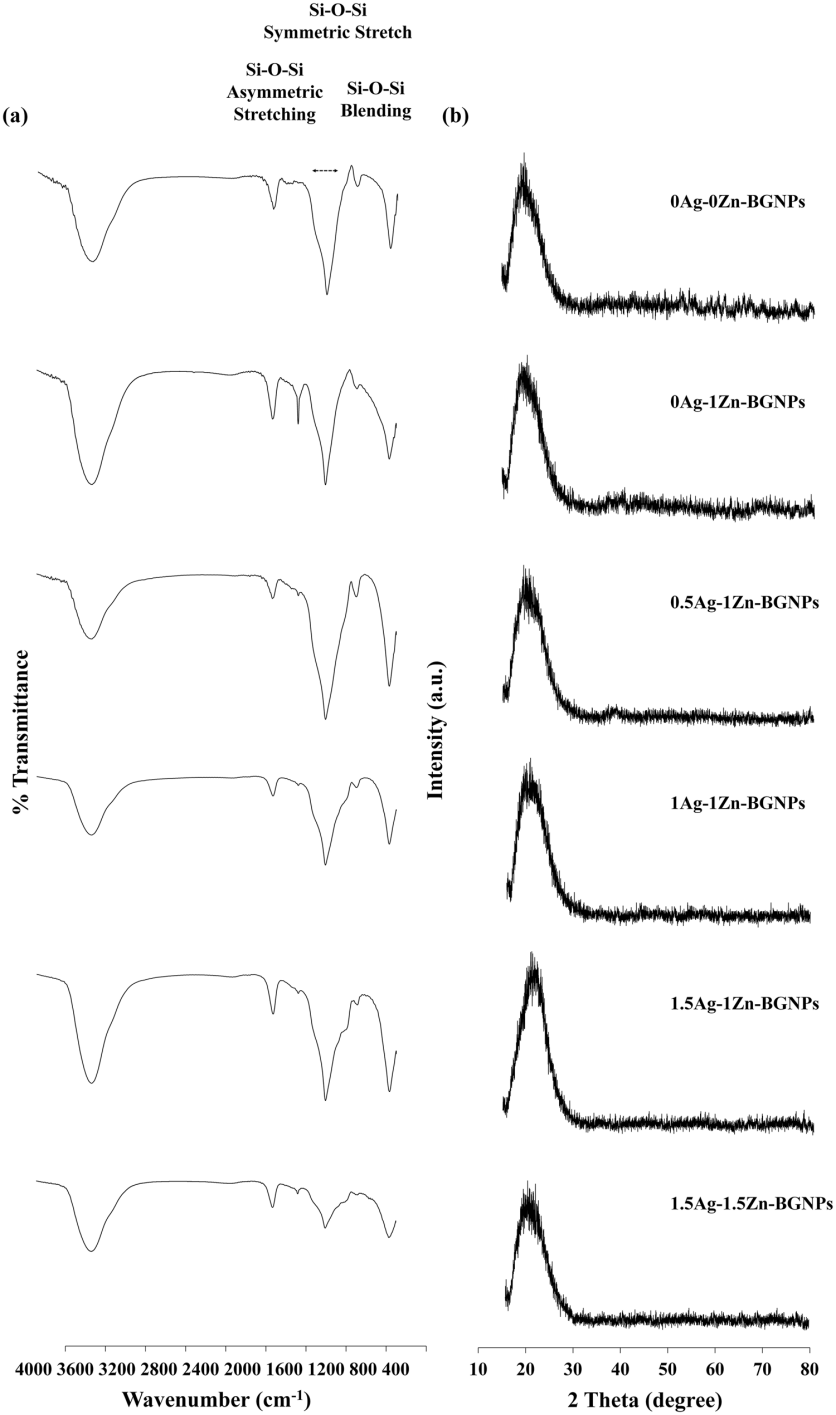
(**a**) FTIR spectra and (**b**) XRD spectra of BGNPs.

ATR-FTIR spectra of BGNPs after calcination at 680 °C and 550 °C showed the unique, characteristic main bands corresponding to the vibration modes of the Si–O–Si bonds in the regions of 1300 and 400 cm^−1^, as shown in Fig. 2a (Left panel). The broad band between 1300 and 1000 cm^−1^ was associated with the Si-O-Si asymmetric stretching vibration. The bands located at approximately 800 cm^−1^ related to the symmetric Si–O–Si stretching, and those at 450 cm^−1^, to the rocking vibration of Si–O–Si bending^30^. The shoulder band at approximately 940 cm^−1^ was reduced when Zn and Ag were doped through the second post-functionalization. These results indicated the formation of bridging oxygen, resulting in an increase in network connectivity of the glass structure. The XRD pattern of typical amorphous BGNPs showed a broad halo at low degrees (around 23–26°), clearly indicating the structural disorder of the glass network prepared using the sol‐gel method (Fig. 2b (Right panel)). The XRD pattern confirmed the completely amorphous nature of all samples. There was no crystal pattern of metal oxide in the XRD results, implying that Zn and Ag were successfully incorporated into the glass network without a structural change^31,32^. Thus, it is possible to tailor BGNPs’ properties by incorporating bivalent cationic ions, including Zn and Ag.

**Figure 3:**
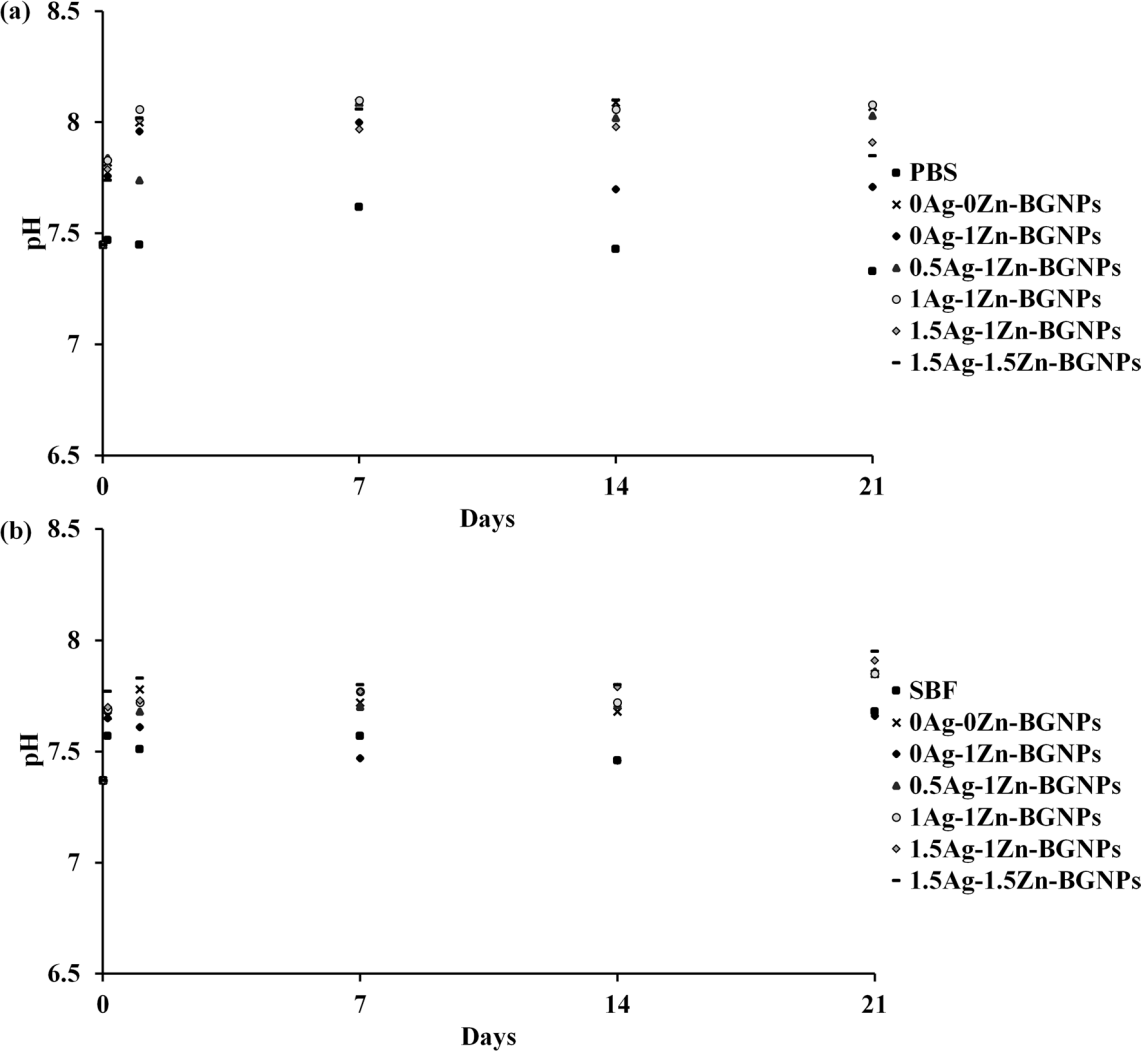
The pH solution of 0Ag–0Zn-BGNPs, 0Ag–1Zn-BGNPs, 0.5Ag–1Zn-BGNPs, 1Ag–1Zn-BGNPs, 1.5Ag–1Zn-BGNPs, and 1.5Ag–1.5Zn-BGNPs in (**a**) PBS solution at pH 7.4 and (**b**) SBF solution at pH 7.4.

The in vitro bioactivity of the prepared dense monodispersed BGNPs was evaluated via apatite formation through immersion in PBS and SBF solution at pH 7.4 in an incubating shaker at 37 °C, shaking at 120 rpm for 4 h, 1 day, 7 days, 14 days, and 21 days of incubation. After the interval time, the pH of the sample solutions was recorded to monitor the hydrolytic stability. The pH of the SBF and PBS solutions for all samples did not significantly change, as shown in Fig. 3a,b. However, the pH solution of 0Ag–0Zn-BGNPs showed a greater increase than other groups. This was because the released ions played a role in stabilizing large changes in the pH of the solutions. The cations in the particle compositions were exchanged with the arouse environment to stabilize silanol groups. The phosphate ions were precipitated on the particles’ surface. These results indicated that the ions released from the particles did not alter the pH solutions over the period of study, implying that the degradation products could not trigger an adverse effect to the cellular environment^20^.

**Figure 4:**
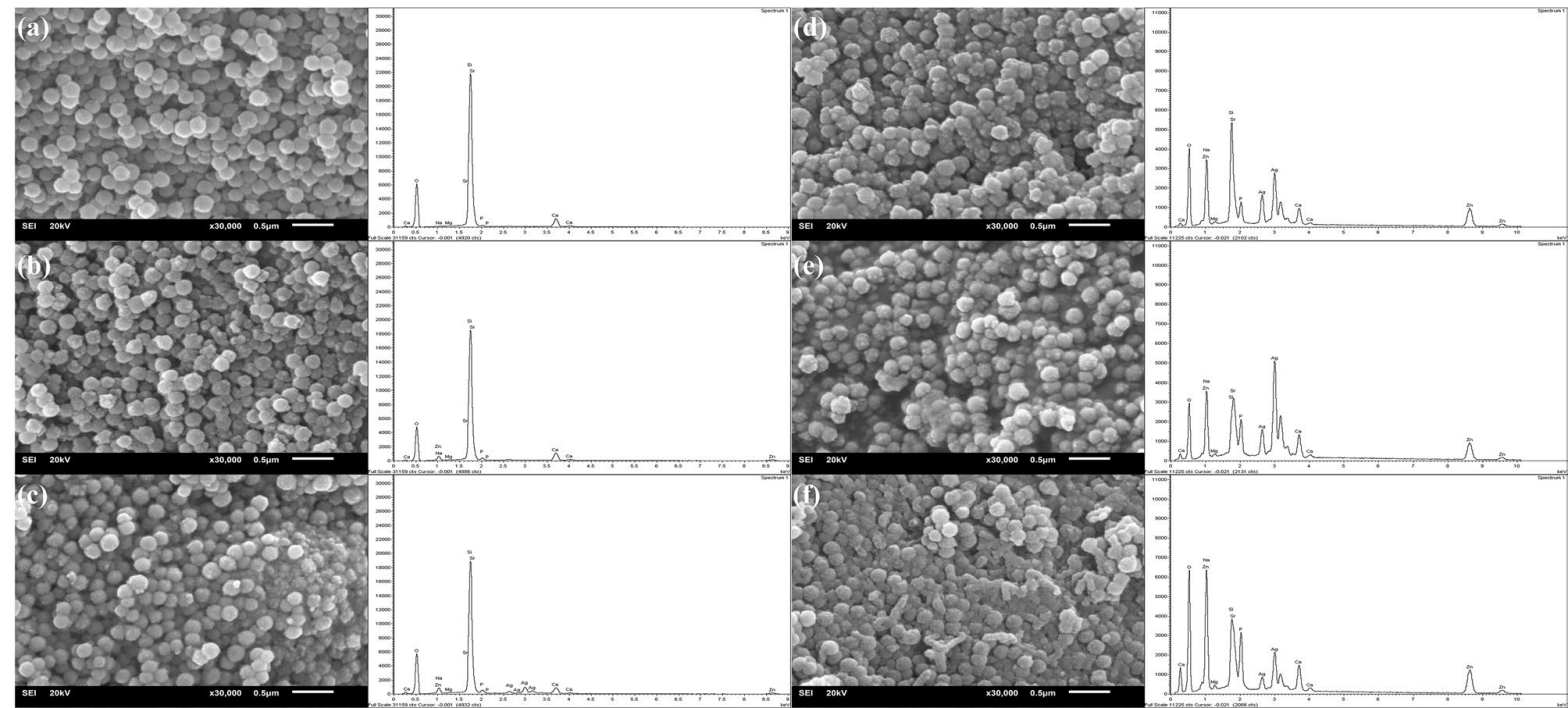
SEM images (Left panel) and EDX-SEM (Right panel) of sol–gel derived BGNPs (**a**) 0Ag–0Zn-BGNPs, (**b**) 0Ag–1Zn-BGNPs, (**c**) 0.5Ag–1Zn-BGNPs, (**d**) 1Ag–1Zn-BGNPs, (**e**) 1.5Ag–1Zn-BGNPs, and (**f**) 1.5Ag–1.5Zn-BGNPs after incubation in the SBF solution for 21 days (operating at 20 kV and magnification 30 kX, scale bars = 500 nm).

**Figure 5:**
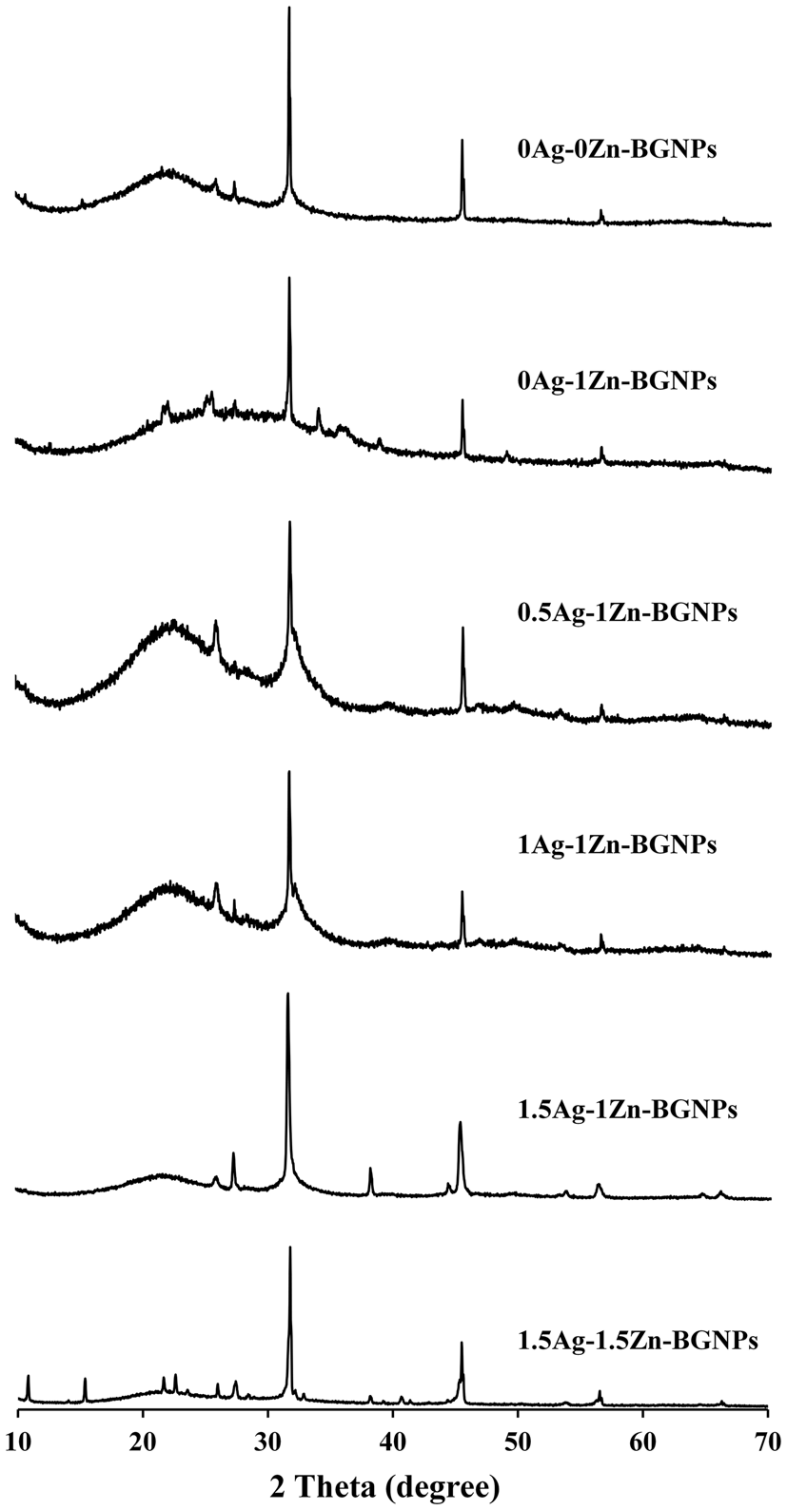
XRD spectra of BGNPs after incubation in the SBF solution for 21 days.

The SEM images of particles after incubation in the SBF solution for 21 days revealed the formation of “cauliflower-like” structures on the particles’ surfaces, as shown in Fig. 4, indicating that apatite deposits had appeared after immersion in the SBF for 21 days. The EDX-SEM profile showed the distribution of Si, Ca, Sr, Ag, Zn, and P in a cross section of BGNPs soaked in SBF. The XRD pattern of particles after incubation in the SBF solution for 21 days confirmed the formation of apatite on the particle surface (Fig. 5). The crystalline peaks were matched with the standard hydroxyapatite (JCPDS card No. 09–0432). The characteristics of major peaks were observed for hydroxyapatite at 2θ = 22.9°, 25.9°, 31.7°, 32.2°, 34.0°, 43.8° and 45.3°. Taken together, the ability of these BGNPs to form an apatite-like layer on the surface and to resorb could increase the bioactivity responsible for the bonding of bioactive glass to the host bone. Substituting Sr for Ca in the nanoparticle composition increased the rate of dissolution and ion release, resulting in the stimulation of apatite formation^33^.

**Figure 6:**
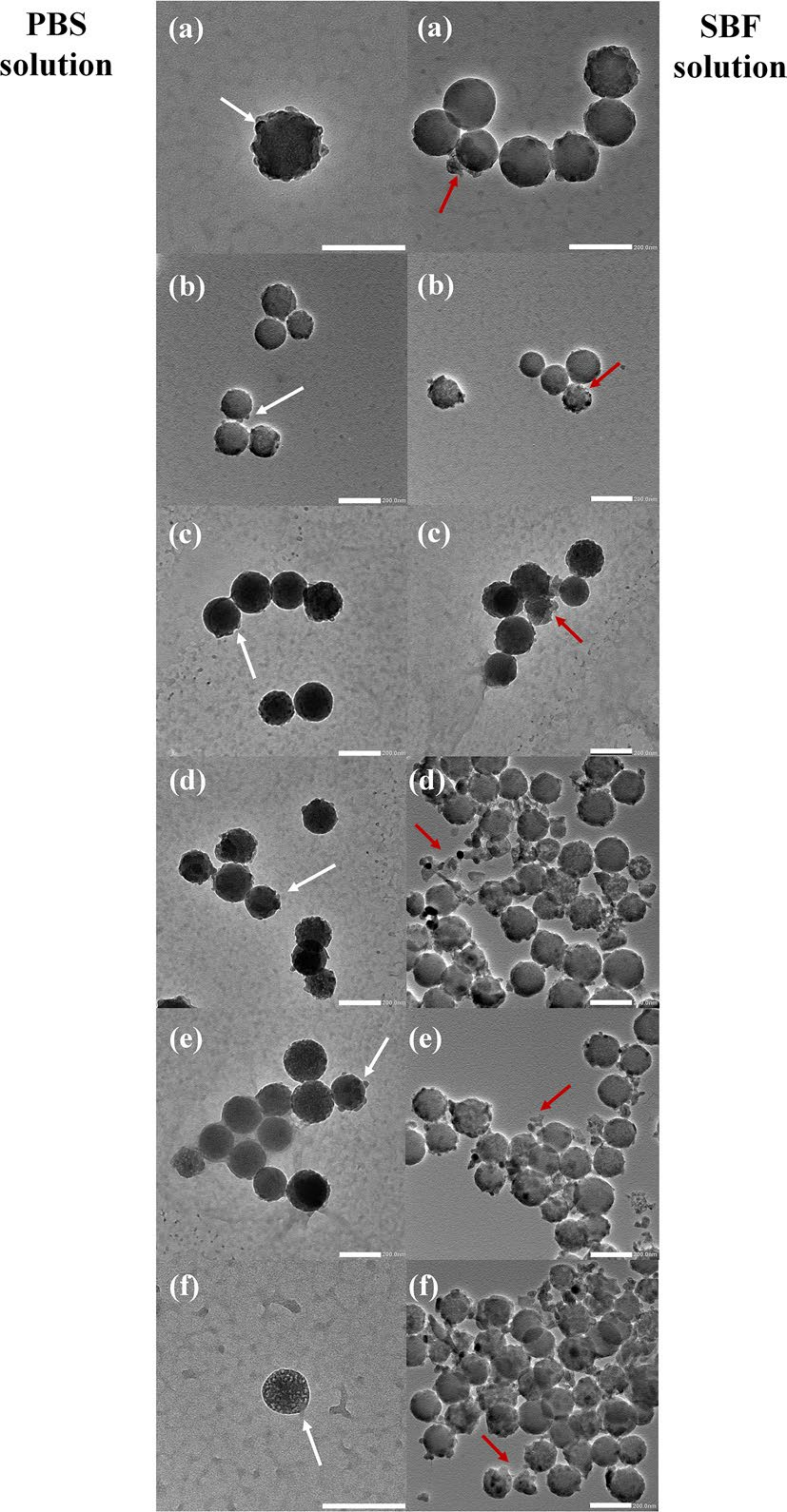
TEM micrographs of (**a**) 0Ag–0Zn-BGNPs, (**b**) 0Ag–1Zn-BGNPs, (**c**) 0.5Ag–1Zn-BGNPs, (**d**) 1Ag–1Zn-BGNPs, (**e**) 1.5Ag–1Zn-BGNPs, and (**f**) 1.5Ag–1.5Zn-BGNPs after incubation in the PBS solution (Left panel) and the SBF solution (Right panel) for 21 days. White arrows indicated the formation of salt on the particle surface in the PBS solution. Red arrows indicated the degraded particles and the apatite formation in the SBF solution. Scale bar: 200 nm.

The bright-field TEM images of particles after immersion in the PBS and SBF solutions for 21 days indicated that the particles had degraded, as shown in Fig. 6. In the PBS solution, there was a small change in the morphology of the particle, from a smooth to rough surface, indicating the formation of salt on the particles’ surface (white arrows in TEM images). In the SBF solution, the reduction in the particle size and the transformation from spherical to irregular shape (red arrows in TEM images) indicated the biodegradation of the particles. These results were correlated with previous studies showing that BGs have the potential to stimulate osteogenesis through the formation of HCA layer, and the ions released from the degradation process^34,35^. Therefore, Zn- and Ag-doped BGNPs have great potential as nanocarriers for delivering therapeutic bivalent cations.

**Figure 7:**
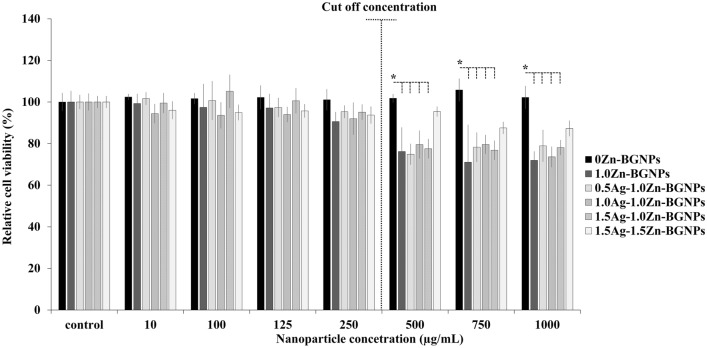
Cell viability of MC3T3-E1 cells exposed to particles which were subtracted from the positive control (cells cultured in nanoparticles-free media). The data are expressed as mean ± SD of three independent experiments (n = 3). (*) indicates a statistically significant difference compared to controls (*p* < *0.05*).

The effect of the particles on cell viability (direct method) against MC3T3-E1 (mouse pre-osteoblast cells), hFOB 1.19 (human fetal osteoblast cells), and MG-63 (human osteosarcoma derived osteoblastic cells) was investigated using MTT assay. Cells were exposed to the particle at a concentration range of 0–1000 µg/mL for 24 h. Untreated cells served as the control. A cell viability of greater than 70% relative cell viability was taken to represent no significant toxicity (ISO 10993-5). Zn-containing BGNPs had no toxicity to the MC3T3-E1 cells up to a concentration of 250 µg/mL, as shown in Fig. 7. At a concentration of 250 µg/mL, 0Ag–1.0Zn-BGNPs, 0.5Ag–1.0Zn-BGNPs, and 1.0Ag–1.0Zn-BGNPs were statistically significantly decreased compared to 0Ag–0Zn-BGNPs.

**Figure 8:**
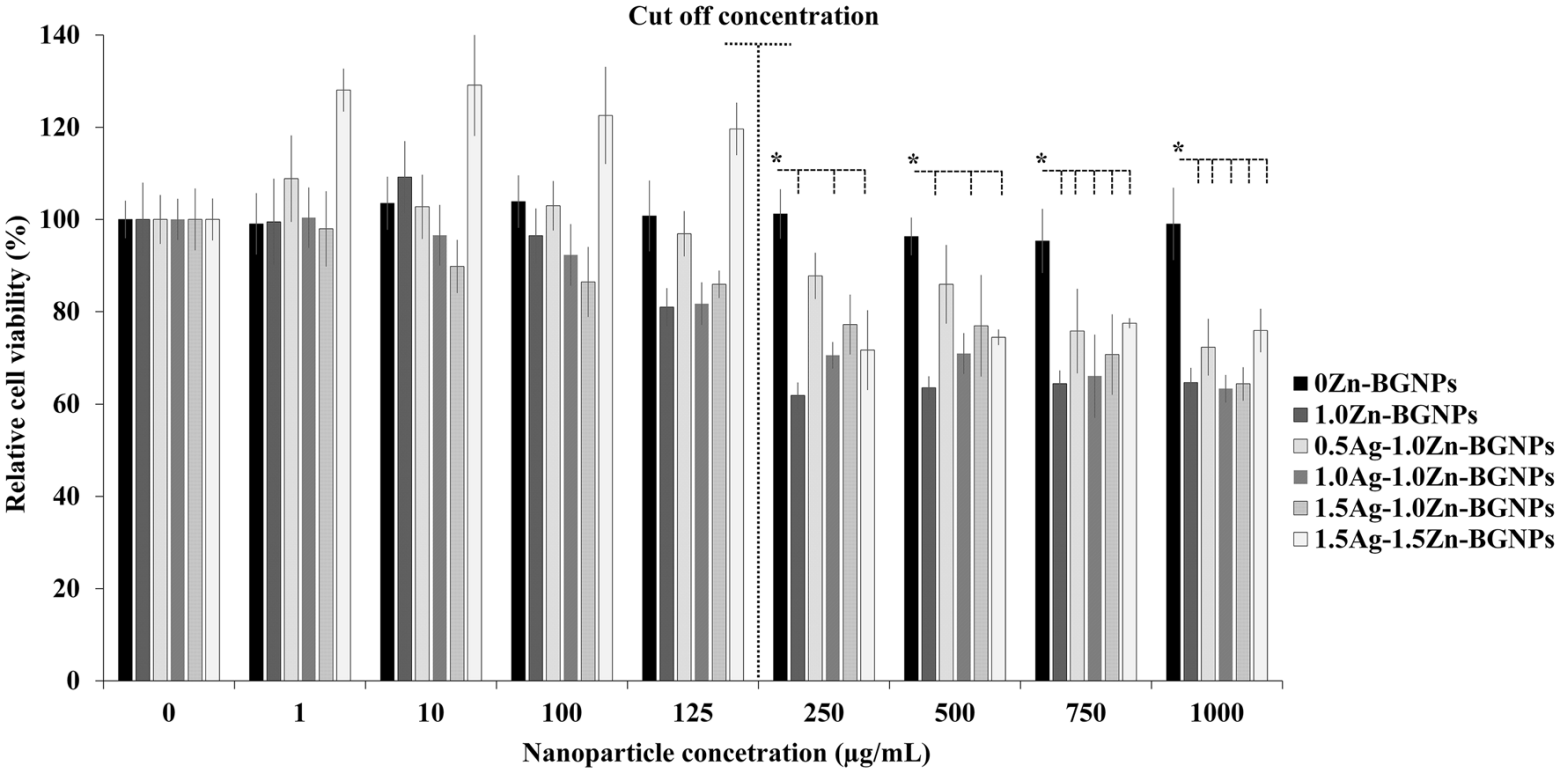
Cell viability of hOB 1.19 cells exposed to particles which were subtracted from the positive control (cells cultured in nanoparticles-free media). The data are expressed as mean ± SD of three independent experiments (n = 3). (*) indicates a statistically significant difference compared to controls (*p* < *0.05*).

For the bone cancer model, to understand whether or not yAg–xZn-BGNPs could be used to kill cancer cells specifically, human bone cancer cells (MG-63) and non-tumor bone cells (hFOB 1.19) were compared. The cell viability of human osteoblast cells treated with particles is shown in Fig. 8. The cell viability of hFOB 1.19 treated with 1.5Ag–1.5Zn-BGNPs was statistically significant up to a concentration of 125 µg/mL (*p* < 0.05). At concentration above 125 µg/mL, the cytotoxicity of hFOB 1.19 treated with Zn- and Ag–containing BGNPs was statistically significantly decreased compared to hFOB 1.19 treated with particles without Zn and Ag (0Ag–0Zn-BGNPs), indicating that yAg–xZn-BGNPs were more toxic against hFOB 1.19 than MC3T3-E1 cells. Thus, the maximum concentration (cut-off level) at which the particles could be used with no toxicity to hFOB 1.19 was 125 µg/mL.

**Figure 9:**
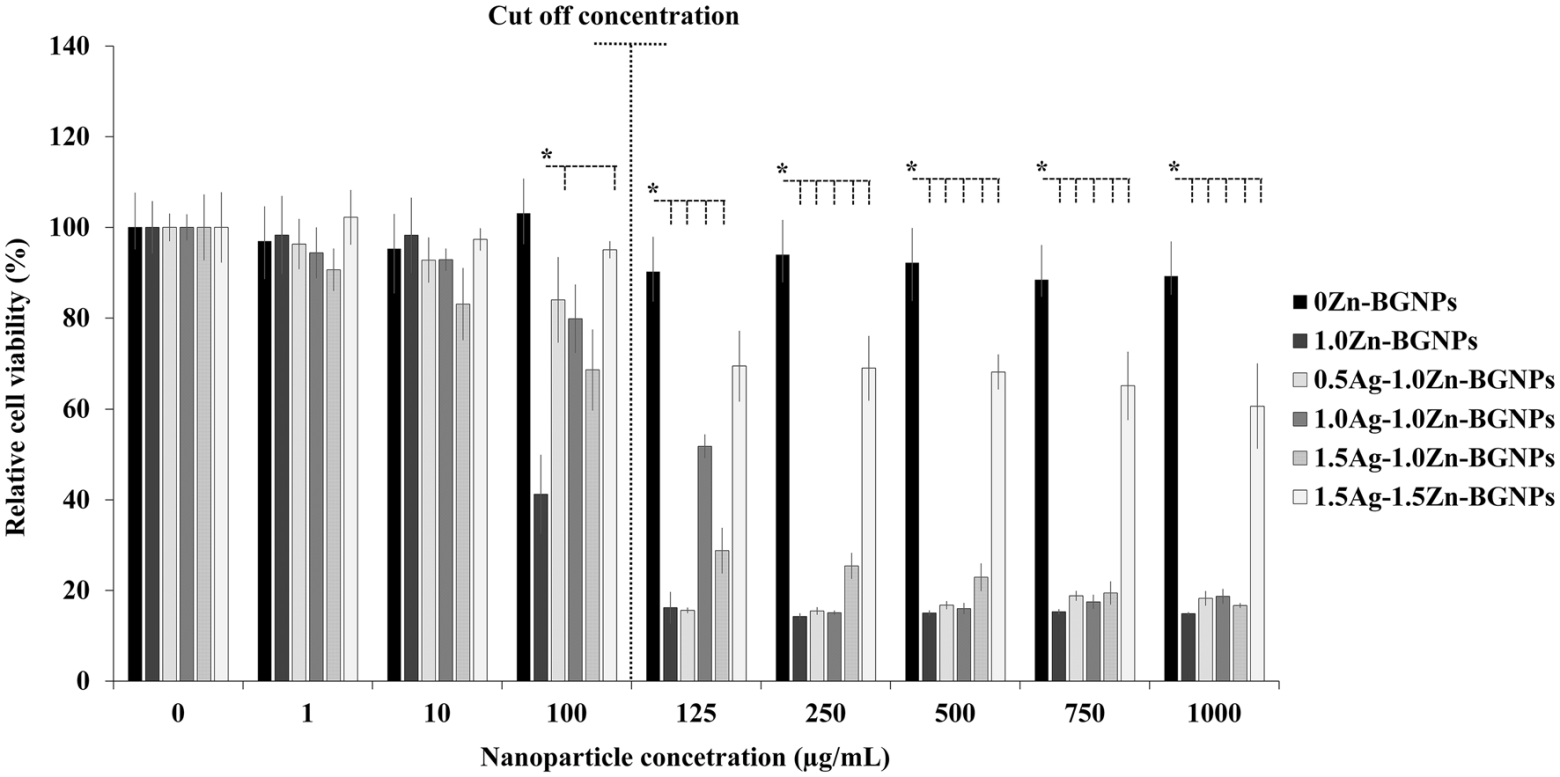
Cell viability of MG-63 cells exposed to particles, which were subtracted from the positive control (cells cultured in nanoparticles-free media). The data are expressed as mean ± SD of three independent experiments (n = 3). (*) indicates a statistically significant difference compared to controls (*p* < *0.05*).

**Figure 10:**
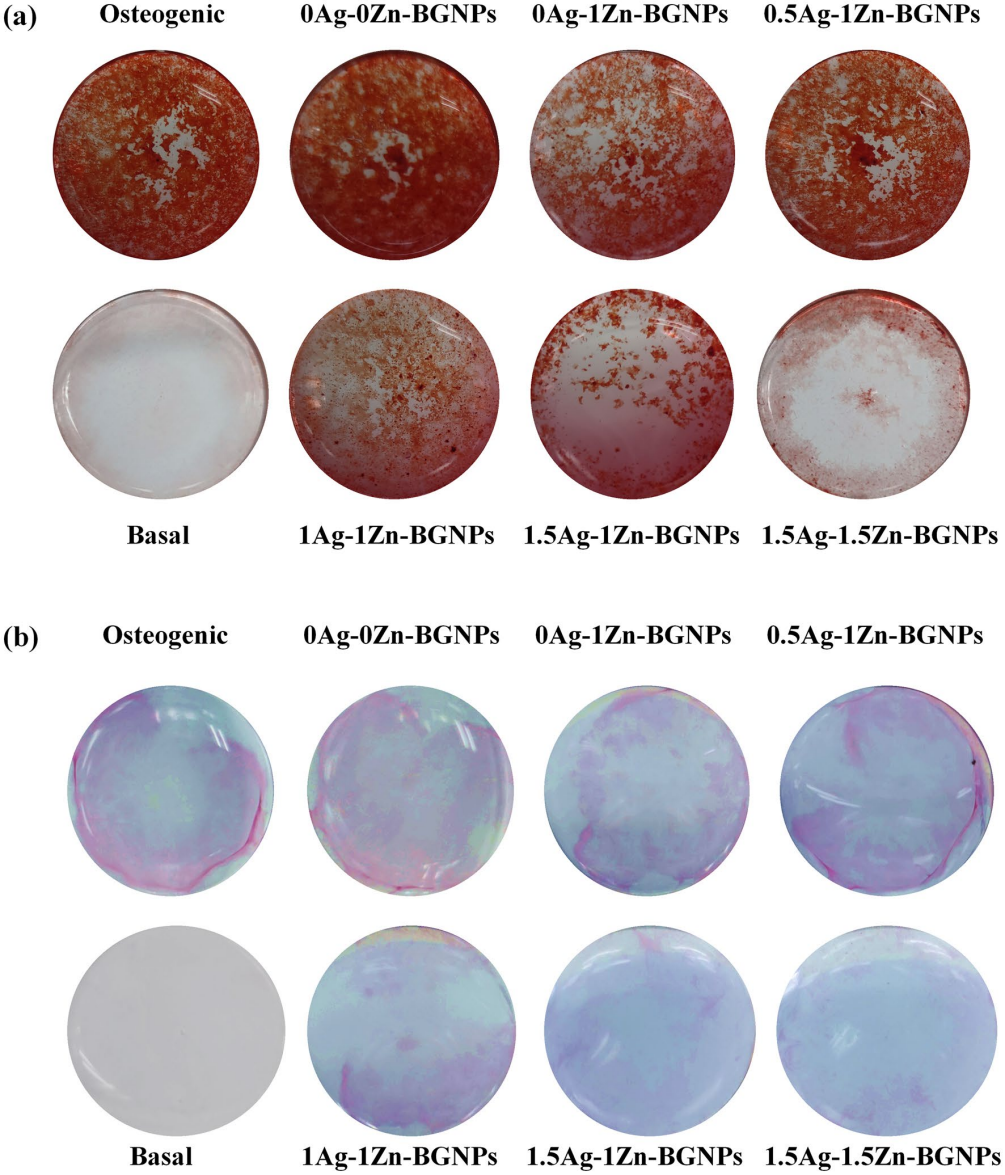
Osteogenesis macroscopic of treated hFOB 1.19 with particles at the concentration of 125 µg/mL (**a**) stained by Alizarin Red S staining and (**b**) stained by ALP activity staining kit following 21 days in culture. The basal and osteogenic media served as the negative and positive control, respectively.

MG-63 (human osteosarcoma derived osteoblastic cells) were exposed to x-Zn-BGNPs for 24 h as shown in Fig. 9. At concentrations up to 10 µg/mL, none of the particles significantly affected the cell viability of MG-63 following 24 h in culture. 0Ag–1ZnBGNPs statistically significantly decreased cell viability at concentrations of ≥ 100 µg/mL (*p* < 0.05). Moreover, 0.5Ag–1Zn‐BGNPs, 1Ag–1Zn‐BGNPs, and 1.5Ag–1Zn‐BGNPs statistically significantly reduced bone cancer cells at concentrations of ≥ 125 µg/mL (*p* < 0.05). These results indicated that 0Ag–1Zn-BGNPs, 0.5Ag–1Zn‐BGNPs, 1Ag–1Zn‐BGNPs, and 1.5Ag–1Zn‐BGNPs at a concentration of 125 µg/mL killed the bone cancer cells (MG-63) preferentially whilst maintaining the cell viability of the normal bone cells (hFOB 1.19). This is because Zn is released from BGNPs in higher amounts under acidic conditions^22,36^. However, 1.5Ag–1.5Zn‐BGNPs have less effectively killed the bone cancer cells resulting from a more compact glass network implying a decrease of ion release. A previous study reported that the intratumorally acidic microenvironment of bone cancer resulted in resorption activity^37^, indicating that Zn was highly released from the yAg–xZn-BGNPs in the bone cancer cells.yAg–xZn-BGNPs could induce osteogenic differentiation of normal bone cells (hFOB 1.19) after 21 days. To investigate the efficiency of osteogenesis, alizarin red S was used to indicate calcified tissue formation (Fig. 10a). The formation of mineralization was significantly increased when the bone cells were exposed to the yAg–xZn-BGNPs at a concentration of 125 µg/mL, especially in 0Ag–0Zn-BGNPs, 0Ag–1Zn-BGNPs, 0.5Ag–1Zn-BGNPs, and 1Ag–1Zn-BGNPs compared to the negative control (untreated cells in the basal condition). The ALP activity of treated cells was significantly observed compared to untreated cells in the basal condition, indicating that yAg–xZn-BGNPs have the capacity to activate osteogenic differentiation in the absence of osteogenic supplements (Fig. 10b). 0Ag–0Zn-BGNPs (46 Si:18 Ca:36 Sr) had a high capacity to induce calcified formation and ALP activity—similarly to the positive control (untreated cells in the osteogenic condition), because Ca and Sr play key functions in bone formation^38,39^. 0Ag–1Zn-BGNPs could induce bone regeneration caused by the function of Ca, Sr, and Zn^22^. An increase in the amount of Ag in the glass composition caused a slight reduction in mineralization precipitation and ALP activity because of the lower amounts of Ca, Sr, and Zn. In this study, Ag was incorporated in BGNPs owing to its remarkable antibacterial property. Therefore, the optimal amount of Ag was important to elucidate. 0.5Ag–1Zn-BGNPs and 1Ag–1Zn-BGNPs provided the effective desired results.

**Figure 11:**
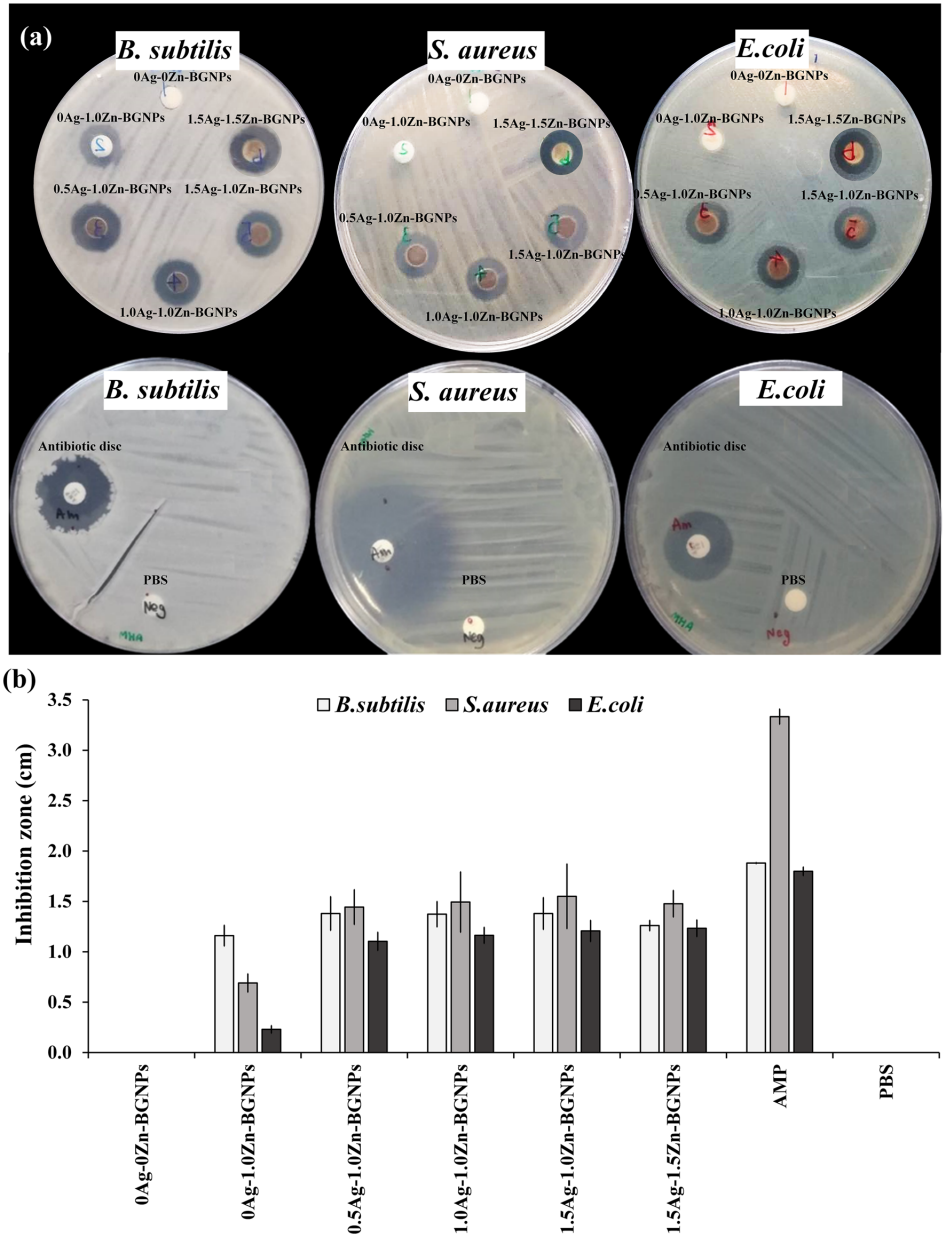
Antibacterial effects assessed using the disc diffusion method against *B. subtilis, S. aureus,* and *E. coli*. (**a**) Antimicrobial diffusion "halo" results, (**b**) the diameters of the inhibition zone (mm) of particles. The data are expressed as mean ± SD of three independent experiments (n = 3). Negative control was PBS and positive control was ampicillin (AMP).

The antibacterial effect of x-Zn-BGNPs was investigated against *B. subtilis*, *S. aureus*, and *E. coli* using a disc diffusion methodology, as shown in Fig. 11a,b. There was no inhibition zone formation for particles without Zn and Ag (0Ag–0Zn-BGNPs). The particles with Zn (0Ag–1Zn‐BGNPs) exhibited an inhibition zone only against *B. subtilis.* The formation of inhibition zones around the paper discs in the agar plates after 16–18 h confirmed the antimicrobial activity of Zn-BGNPs containing Ag (0.5Ag–1Zn-BGNPs, 1Ag–1Zn-BGNPs, 1.5Ag–1Zn-BGNPs, and 1.5Ag–1.5Zn-BGNPs) against *B. subtilis*, *S. aureus*, and *E. coli* (Fig. 11a). For the same type of bacteria, the antibacterial effect of 0Ag–1Zn‐BGNPs, 0.5Ag–1Zn‐BGNPs, 1Ag–1Zn‐BGNPs, 1.5Ag–1Zn‐BGNPs, and 1.5Ag–1.5Zn-BGNPs against *B. subtilis* was significantly increased compared to the negative control (PBS). Ag–doped Zn-BGNPs (0.5Ag–1Zn‐BGNPs, 1Ag–1Zn‐BGNPs, 1.5Ag–1Zn‐BGNPs, and 1.5Ag–1.5Zn‐BGNPs) showed large inhibition zones against *S. aureus* and *E. coli* compared to the control. 0.5Ag–1Zn‐BGNPs, 1Ag–1Zn‐BGNPs, 1.5Ag–1Zn‐BGNPs, and 1.5Ag–1.5Zn‐BGNPs had an antibacterial effect against Gram-positive and Gram-negative bacteria that could have been facilitated by Ag’s DNA replication-destroying function^40^.The preliminary results confirmed that Ag enhanced the antibacterial effects and assisted the Zn activity against bacterial growth. The possible mechanism of yAg–xZn‐BGNPs to inhibit bacterial growth was that Zn and Ag ions were released through the degradation of yAg–xZn‐BGNPs. Then they penetrated across cell membrane and generated reactive oxygen species (ROS) inside the cell^41^.

## Conclusion

Multifunctional Zn and Ag co-doped BGNPs with a diameter range of 150 ± 30 nm were synthesized through the sol–gel and two-step post-functionalization processes. The incorporation of Zn and/or Ag, a network modifier, improved the bioactivity of BGNPs without altering the particle size and morphology. The amorphous nature of the synthesized particles could enhance bioactivity through apatite formation and by increasing resorption. Moreover, the modified particles stimulated calcium deposition and ALP activity, which were the key osteogenic differentiation markers. Particles co-doped with Zn and Ag had the ability to inhibit metabolic activity of the bone cancer cells (MG-63) preferentially whilst maintaining the cell viability of the normal bone cells (hFOB 1.19 and MC3T3-E1) compared to the particle without Zn and Ag. Thus, Zn released from the particles presented excellent anticancer activity. Ag–doped Zn-BGNPs exhibited an anti-bacterial effect against Gram-positive and Gram-negative bacteria while the particles doped with only Zn (0Ag–1.0Zn‐BGNPs) did not show anti-bacterial activity. Thus, Ag exhibited strong antibacterial ability against *S. aureus*, and *E. coli.* To integrate the ability to stimulate bone regeneration, exhibit anticancer, and antibacterial activity into the BGNPs, Zn and Ag co-doped BGNPs (0.5Ag–1Zn‐BGNPs and 1Ag–1Zn-BGNPs) are promising biomaterials for the treatment of tumor-induced bone defects. These multi-functional nanoparticles can be applied to use as filler composite scaffold or coating materials for bone tissue engineering.

## Methods

All reagents were from Sigma–Aldrich (Thailand) unless stated otherwise. Ethyl alcohol (99.5%), ammonium hydroxide, tetraethyl orthosilicate (TEOS), calcium nitrate tetrahydrate (99%), strontium nitrate (99%), zinc nitrate hexahydrate (≥98%), silver nitrate (99%), phosphate buffered saline (PBS), sodium chloride (NaCl), sodium hydrogen carbonate (Na-HCO_3_), potassium chloride (KCl), di-potassium hydrogen phosphate trihydrate (K_2_HPO_4_.3H_2_O), magnesium chloride hexahydrate (MgCl_2_.6H_2_O), hydrochloric acid (HCl), calcium chloride (CaCl_2_), sodium sulfate (Na_2_SO_4_), nitric acid, Eagle’s Minimum Essential Medium (EMEM, Gibco^TM^) minimum essential medium eagle alpha (α-MEM, Gibco^TM^), Ham’s F12 Medium Dulbecco’s Modified Eagle’s Medium (Gibco^TM^), fetal bovine serum (FBS, Thermo Fisher Scientific), Antibiotic-Antimycotic (Thermo Fisher Scientific), trypsin-EDTA (Thermo Fisher Scientific), minimum essential medium eagle alpha modification (α-MEM) with nucleosides (Gibco^TM^), sodium bicarbonate (NaHCO_3_), 3-(4,5-dimethylthiazol-2-yl)-2,5-diphenyltetrazolium bromide (MTT, Thermo Fisher Scientific), dimethyl sulfoxide (DMSO), dexamethasone (DEX), β-glycerophosphate, ascorbic acid, paraformaldehyde, Alizarin Red S, Mueller-Hinton Agar (MHA, Difco™). MC3T3-E1 cells (ATCC® CRL-2593™), MG-63 cells (ATCC® CRL-1427™), and hFOB 1.19 cells (ATCC® CRL-11372™) were purchased from ATCC (Biomedia, distributor of ATCC).

### Zn- and Ag–doped sol–gel-derived BGNP synthesis

BGNPs were synthesized using the sol–gel process described in our previous work^13^. Dense silica nanoparticles (SiO_2_‐NPs) with a diameter range of 150 ± 30 nm were synthesized. Briefly, 5.8 mL of ammonium hydroxide, 49.7 mL of Milli-Q water, and 390 mL of ethyl alcohol (99.5%) were mixed in a 1 L Erlenmeyer flask with a stirring rate of 600 rpm for 15 min. Then, 30 mL of tetraethyl orthosilicate (TEOS) was added into the prepared solution and stirred overnight to complete the hydrolysis and condensation reactions that occurred simultaneously to form the silica network (Si–O–Si). A white colloidal solution of SiO_2_-NPs was centrifuged at 5000 rpm for 40 min to collect SiO_2_-NPs, and then washed with ethanol (two times) and distilled water (one time) to remove unreacted substances.

**Figure 12:**
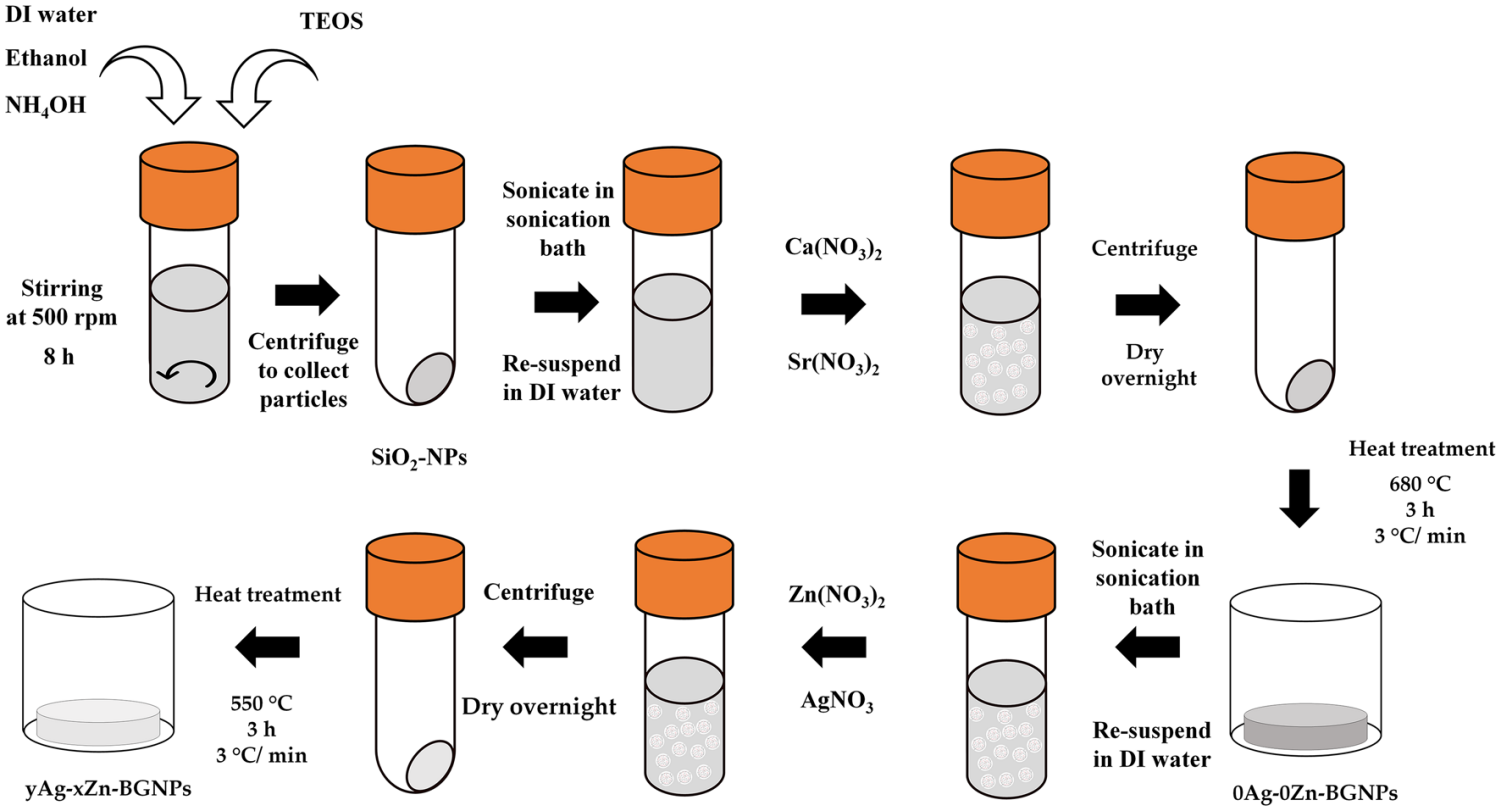
Synthesis process of Zn- and Ag–doped sol–gel-derived BGNP.

The SiO_2_‐NPs were incorporated with Ca^2^ (calcium nitrate tetrahydrate) and Sr^2+^ (strontium nitrate) at a nominal ratio of 1.0 SiO_2_:0.5 CaO:1.5 SrO. Particles were dried at 60 °C in the oven overnight before calcination at 680 °C for 3 h at a heating rate of 3 °C /min to remove nitrate precursors and obtain Sr-containing BGNPs. After that, BGNPs were second-doped with Zn^2+^ (zinc nitrate hexahydrate) and Ag^+^ (Silver nitrate) at the nominal ratio of 1.0 SiO_2_:0.5 CaO:1.5 SrO:x Zn:y Ag, where x = 0, 1, and 1.5 and y = 0, 0.5, 1.0, and 1.5, through second-step post functionalization as shown in Table 3. The doped particles were dried and heated at 550 °C for 3 h at a heating rate of 3 °C /min to obtain yAg–xZn-BGNPs (Fig. 12). Finally, these particles were washed with ethanol to remove excess Ca, Sr, Zn, and Ag that had not been incorporated into the particles. The cleaned particles were dried at 60 °C overnight.

**Table 3:** Compositions of bioactive glass nanoparticle (nominal ratio).

	Nominal ration				
	Si	Ca	Sr	Zn	Ag
0Ag–0Zn-BGNPs	1.0	0.5	1.5		
0Ag–1Zn-BGNPs	1.0	0.5	1.5	1.0	
0.5Ag–1Zn-BGNPs	1.0	0.5	1.5	1.0	0.5
1Ag–1Zn-BGNPs	1.0	0.5	1.5	1.0	1.0
1.5Ag–1Zn-BGNPs	1.0	0.5	1.5	1.0	1.5
1.5Ag–1.5Zn-BGNPs	1.0	0.5	1.5	1.5	1.5

### BGNP characterization

The particle size and surface charge of the BGNPs were measured using Nanosizer (Horiba; SZ-100V2). The particles were suspended in DI water. Three measurements were obtained for each suspension and average value was reported with standard deviation. The surface morphology the BGNPs was imaged using a Scanning Electron Microscope (SEM) operating at 20 kV. An X-ray diffractometer (XRD) was used to identify the crystallized pattern of the particles. The XRD pattern was collected with a Bruker AXS automated powder diffractometer using Cu Kα radiation (1.540600 A°) at 40KV/40mA. Data were collected in the 10–70° 2θ range with a step size of 0.02° and a dwell time of 1.0 s. Fourier transform infrared spectroscopy (FTIR; Thermo Scientific Nicolet iS5) was used in attenuated total reflection (ATR) mode at a wavenumber ranging from 4000 to 400 cm^−1^, at a scan speed of 32 scan/min, and with a resolution of 4 cm^−1^. To determine the elemental composition of BGNPs, X-ray fluorescence (XRF: Fischer/XUV773) operated in a vacuum with X-ray generators in the 8–20 kV range was used.

### Bioactivity assessment

To compare the release profile of ions from the yAg–xZn-BGNPs, the release of Si, Ca, Sr, Zn, and Ag ions from yAg–xZn-BGNPs was monitored as a function of time for 21 days in two different solutions: simulated body fluid (SBF) at pH 7.4, and phosphate-buffered saline (PBS) at pH 7.4. The SBF solution was prepared following a previous study^42^. The SBF had to be pre-heated at 37 °C and pH-adjusted to 7.40 before starting the experiment. The *in vitro* bioactivity assessment was conducted by incubating 75 mg of yAg–xZn-BGNPs in the 50 mL of SBF and PBS solutions at pH 7.4 and 37 °C, with shaking at 120 rpm for 21 days. At the end of the incubation period, the yAg–xZn-BGNPs were collected by centrifugation and then immediately washed with ethanol and subsequently with acetone to terminate the reactions. The morphological structure of yAg–xZn-BGNPs was characterized using SEM and TEM.

### Cell culture

MC3T3-E1 cells (ATCC^®^ CRL-2593™) of the murine pre-osteoblast cell line were routinely cultured in a T-75 flask under standard conditions in a humidified atmosphere at 37 °C and 5% CO_2_ in α-MEM (Thermo Fisher Scientific) supplemented with 10% fetal bovine serum (FBS, Thermo Fisher Scientific) (v/v), 100 U/mL Antibiotic-Antimycotic (Thermo Fisher Scientific). Cells were passaged by trypsinizing using trypsin-EDTA (500 μg/mL) (Thermo Fisher Scientific) upon confluence (70–80%) and re-suspended in the α-MEM before cells were counted. The cell stock was diluted to the desired concentration (5 × 10^5^ cells/mL).

MG-63 cells (ATCC^®^ CRL-1427™) of the human osteosarcoma cell line were routinely cultured in a T-75 flask under standard conditions in a humidified atmosphere at 37 °C and 5% CO_2_ in Eagle’s Minimum Essential Medium (EMEM) (ATCC, 30-2003™) supplemented with 10% fetal bovine serum (FBS, Thermo Fisher Scientific) (v/v), 100 U/mL Antibiotic-Antimycotic (Thermo Fisher Scientific). Cells were passaged by trypsinizing using trypsin-EDTA (500 μg/mL) (Thermo Fisher Scientific) upon confluence (70-80%) and re-suspended in the EMEM before cells were counted. The cell stock was diluted to the desired concentration (5 × 10^5^ cells/mL).hFOB 1.19 cells (ATCC^®^ CRL-11372™) of the human fetal osteoblast cell line were routinely cultured in a T-75 flask under standard conditions in a humidified atmosphere at 37 °C and 5% CO_2_ in a 1:1 mixture of Ham’s F12 Medium Dulbecco’s Modified Eagle’s Medium, with 2.5 mM L-glutamine (without phenol red), supplemented with 10% fetal bovine serum (FBS, Thermo Fisher Scientific) (v/v), 100 U/mL Antibiotic-Antimycotic (Thermo Fisher Scientific), and 0.3 mg/mL G418.

### Cell viability

To evaluate the cytotoxicity effect of yAg–xZn-BGNPs, cell viability was measured using MTT colorimetric assay (Thermo Fisher Scientific) according to the manufacturer’s instructions. MC3T3-E1, MG-63, and hFOB 1.19 cells were seeded in flat-bottomed 96-well plates (Corning) with a cell concentration of 5 × 10^3^ cells/well. The cells were incubated at 37 °C overnight to allow the cells to attach in a monolayer. After that, the cell culture media was replaced with media containing NPs at concentrations ranging from 0–1 mg/mL: namely 0, 10, 100, 125, 250, 500, 750, and 1000 µg/mL. Cells were exposed to particles for 1 day (direct contact). The control comprised cells without particle exposure. Cell viability was determined using the MTT colorimetric assay based on the conversion of 3-(4,5-dimethylthiazol-2-yl)-2,5-diphenyltetrazolium bromide (MTT) into formazan. Formazan is soluble in dimethyl sulfoxide (DMSO), and the concentration of soluble formazan was determined using a microplate reader (Infinite^®^ 200 Tecan, Austria) at 570 nm. The relative cell viability (% viability compared to the control, i.e., the untreated cells with the particles) was calculated as a mean value ± standard error of the mean.

### Osteogenic differentiation

To evaluate the osteogenic differentiation of hFOB 1.19 cells, alkaline phosphatase (ALP) activity and alizarin red s were monitored. hFOB 1.19 cells in the basal medium were seeded in flat-bottom 48-well plates with a cell concentration of 2 × 10^3^ cells/well. The cells were incubated at 37 °C overnight to allow the cells to attach in a monolayer. The osteogenic medium served as the positive control. The basal medium was supplemented with 10 nM dexamethasone (DEX, Sigma-Aldrich), 10 mM β-glycerophosphate (Sigma-Aldrich), and 100 µg/mL ascorbic acid (Sigma-Aldrich). After that, the cell culture media was replaced with media containing NPs at a concentration of 125 µg/mL. Cell culture media were routinely changed twice a week for 21 days. On the 21st day in culture, osteoblastic differentiation of hFOB 1.19 cells was measured via cellular ALP activity staining (Abcam) according to the manufacturer’s instructions. Calcium phosphate deposits were detected. The cells were fixed with 4% paraformaldehyde and stained with 2% Alizarin Red S in PBS at pH 4.2 to detect calcified tissue formation.

### Antibacterial activity

Antibacterial activity was determined using the disc diffusion method. *Bacillus subtilis* ATCC 6633, *Staphylococcus aureus* ATCC 25923, and *Escherichia coli* ATCC 25922 isolates were grown overnight on Mueller Hinton Agar (MHA). Direct colony suspensions were prepared in sterile saline to achieve a turbidity equivalent to 0.5 McFarland standards. The inoculum suspensions were streaked onto the dried surface of an MHA plate with a sterile cotton swab and left to absorb for no more than 15 minutes. The particles were re-suspended in the PBS solution at the concentration of 250 µg/mL in the ultrasonication bath for 30 minutes. Twenty microliters of BGNPs were pipetted onto 6-mm-diameter Whatman^®^ antibiotic assay discs in a sterile dish. PBS and antibiotic (ampicillin, AMP 10 µg, Thermo Scientific™) was served as the negative and positive control, respectively. Discs were placed aseptically onto the plates immediately. The plates were incubated at 37 °C for 16 to 18 h and the diameter of the inhibition zones or halo zones was measured using a sliding caliper.

### Statistical analysis

Statistical analyses were performed by one-way analysis of variance (ANOVA) in Minitab. A *p* value < 0.05 was considered significant. The graphs shown present the results as the mean values, with the standard deviations (SD) as the error bars. All the quantitative experiments were carried out at least in triplicate.

## Data Availability

The datasets used and/or analyzed during the current study available from the corresponding author on reasonable request.
